# Supplementary material for: Comparison of Micronutrient Intervention Strategies in Ghana and Benin to Cover Micronutrient Needs: Simulation of Bene-Fits and Risks in Women of Reproductive Age
Source: Nutrients. 2021 Jul 1;13(7):2286. doi: 10.3390/nu13072286 (PMC8308306; doi:10.3390/nu13072286)
Supplement: Supplementary file 1 [file nutrients-13-02286-s001.zip › nutrients-1254135-supplementary.pdf]

nutrients

MDPI

Supplementary table S1-a: Scenarios of basic diet for Benin

	Rural women				Urban women			
	non-pregnant, non-breastfeeding		pregnant or breastfeeding		non-pregnant, non-breastfeeding		pregnant or breastfeeding	
	Dishes	Quantity (g/person)	Dishes	Quantity (g/person)	Dishes	Quantity (g/person)	Dishes	Quantity (g/person)
Breakfast	Aklui: mawè gruel	400	Aklui: mawè gruel	400	White bread	87,5	White bread	87,5
	Palmnut sauce	125	Palmnut sauce	125	Margarine	15	Margarine	15
			Fried tomato with vegetable oil	100				
Snacks morning					Wheat porridge	180	Wheat porridge	100
							Soya milk	100
Lunch					Atassi	395	Atassi	395
					Fried tomato with vegetable oil	55	Fried tomato with vegetable oil	55
Snacks afternoon	Eba: cassava dough	100	Eba: cassava dough	100	Doko (wheat fried dough)	140	Doko	140
	Moyo sauce	40	Moyo sauce	40			Watermelon	100
Dinner	Atassi	400	Atassi	400	Maize dough	452	Maize dough	450
	Fried tomato with vegetable oil	200	Fried tomato with vegetable oil	100	Goman sauce	205	Goman sauce	205
			Palmnut sauce	125				
Snacks evening	Eba: cassava dough	100	Eba: cassava dough	100	Tomato soup	125	Atta	40
	Moyo sauce	40	Moyo sauce	40				
			Fried tomato with vegetable oil	100				

Supplementary table S1-b: Scenarios of basic diet for Ghana

	Rural women: non-pregnant, non-breastfeeding		Rural women: pregnant or breastfeeding		Urban women: non-pregnant, non-breastfeeding		Urban women: pregnant or breastfeeding	
	**Dishes**	**Quantity (g/person)**	**Dishes**	**Quantity (g/person)**	**Dishes**	**Quantity (g/person)**	**Dishes**	**Quantity (g/person)**
**Breakfast**	Waakye (rice and cowpeas)	120	Waakye	220	Brown bread	87,5	White bread	87,5
	Fish stew	80	Palmnut soup*	125	Margarine	5	Margarine	5
	Tea	80	Tea	80	Fried eggs	80	Tea	80
	White rice	87,5			Tea	80	Milk	20
					Milk	20		
**Lunch**					Kenkey* (cooked balls of fermented maize dough)	185	Kenkey*	200
					Pepper sauce	80	Pepper sauce	80
**Dinner**	Jollof rice	220	Rice ball	220	White rice	185	Kenkey*	200
			Palmnut soup*	125	Tomato stew	80	Fish okro stew	80
**Snacks**	Corn porridge*	220	Corn porridge*	220	Pineapple	150	Soya milk	250
	Fried yam**	50	Orange**	100	Roasted groundnuts**	50	Watermelon**	100
							Corn porridge*	125
							Ice cream	40

**Basic maize composition was changed for biofortified maize in the scenarios including biofortification*

**** replaced by fried biofortified sweet potatoes in the scenarios including biofortification**

nutrients MDPI

Supplementary table S2: Estimated average requirements (EAR) , recommended nutrients intakes (RNI) and tolerable upper limits (UL) set by WHO for micronutrients

		Fe (mg)	Zn (mg)	Vit A-RAE (µg)	Niacin (mg)	Folate (µg)	Iodine (µg)
**Women of reproductive age**	**EAR**	29,4	4,1	357	11	320	107
	**RDA or RNI**	58,8	4,9	500	14	400	150
	**UL**	45	45	3000	35	1000	1100
**Pregnant or lactating women**	**EAR**	40	5,8	571	14	480	143
	**RDA or RNI**	30	7	800	18	600	200
	**UL**	(45)*	45	3000	35	1000	2600

*WHO doesn't set UL for iron. 45mg of iron/ day is the UL set by IoM.
